# Endocrine disruptors and spontaneous premature labor: a case control study

**DOI:** 10.1186/1476-069X-6-35

**Published:** 2007-11-15

**Authors:** Stephen L Wood, John J Jarrell, Cheryl Swaby, Sui Chan

**Affiliations:** 1 Department of Obstetrics and Gynecology and Community Health Sciences. University of Calgary. 1403 29^th ^St NW Calgary Alberta Canada; 2 Department of Obstetrics and Gynecology University of Calgary. 1403 29^th ^St NW Calgary Alberta Canada; 3 Department of Pharmacology and Therapeutics University of Calgary. 3330 Hospital Drive NW, Calgary Alberta Canada

## Abstract

**Background:**

Premature labor is a poorly understood condition. Estrogen is thought to play a key role and therefore the labor process may be affected by endocrine disruptors. We sought to determine whether or not an environmental toxicant, DDE, or dietary derived endocrine disruptors, daidzein and genistein, are associated with spontaneous preterm labor.

**Methods:**

Cases were defined as primiparous patients having a preterm delivery at or before 35 weeks following the spontaneous onset of labor. Controls were defined as primiparous women who delivered on the same day as the cases but at term gestation.

Over approximately 1 year, 26 cases and 52 controls were recruited. Subjects agreed to have blood tests on day one postpartum for DDE and for the phytoestrogens genistein and daidzein.

**Results:**

The mean concentration of DDE was similar in the case and control groups: 4.29 vs 4.32 ng/g lipid p = .85. In the case group, 13/26 had detectable levels of daidzein (range 0.20 – 1.56 ng/ml) compared to 25/52 controls (range 0.21 – 3.26 ng/ml). The mean concentration of daidzein was similar in cases compared to controls: 0.30 vs .34 ng/ml p = 0.91. Of the case group,14/26 had detectable levels of genistein (range 0.20 – 2.19 ng/ml) compared to 32/52 controls (range 0.21 – 2.55 ng/ml). The mean concentration of genistein was similar in cases compared to controls: 0.39 vs 0.31 ng/ml, p = 0.61.

**Conclusion:**

The serum levels of DDE in this population were found to be low.

There appears to be no relationship between serum concentrations of DDE, daidzein, and genistein and spontaneous preterm labor in our population. The inability to identify an effect may be related to the comparatively low concentrations of DDE in our population and the rapid and variable reduction of phytoestrogens from women in labor.

## Background

Premature delivery occurs after spontaneous labor in approximately 6% of pregnancies (<37 weeks gestation) and remains the single, most important, cause of perinatal mortality and morbidity [1,2]. The pathophysiology of preterm labor is not entirely clear but reproductive hormones, such as progesterone and estrogen appear to have a role. Progesterone is widely regarded as promoting uterine quiescence. Estrogen, on the other hand, may promote myometrial activation with increased receptivity to uterotonic agents by up regulating membrane receptors and gap junctions[3]. Therefore, endocrine disruptors, especially those that have estrogen like effects, may have a role in preterm labor.

The phytoestrogens are of increasing interest owing to their estrogenic properties and ubiquitous exposure from a variety of foods and the ability of individuals to alter the exposure through soy supplementation [4]. These chemicals have been shown to exert estrogenicity through binding to the ErB receptor. With regard to pregnancy, genistein and daidzein have been isolated in amniotic fluid with evidence of transfer from the mother to the fetus [5,6]. Phytoestrogens have also been associated with regulation of the HOX genes that are particularly of interest in pregnancy [7,8] and have been shown to reduce the release of HCG from human placentas at term in vitro [9]. Furthermore, the oral administration of phytoestrogens have been reported to reverse the anti-estrogenic actions of clomiphene citrate on the endometrial lining [10].

DDT and PCBs have also been of interest in the pathogenesis of premature labor and have shown to increase myometrial contractility in-vitro [11-13]. A large, retrospective, U.S., cohort study reported an association between maternal serum DDE, the metabolite of DDT, and premature delivery [14]. However, the serum samples were from women delivering in the 1960's when DDT use was much more prevalent. A subsequent small, case control study of patients in New York found no relationship between serum DDE levels and premature delivery [15]. The phytoestrogens, genistein and daidzein, are also of interest. These chemicals have inherent estrogenic activity on the Erα and Erβ receptors and have been isolated in human amniotic fluid [16]. Previous work at our center had documented significant levels of the DDT metabolite DDE in maternal serum in a cross sectional survey [17]. In addition, in this cross sectional survey, a significant number of women were also found to have detectable levels of the phytoestrogens daidzein and genistein in both serum samples and in amniotic fluid [18]. Furthermore, there was a strong correlation of serum and amniotic fluid concentrations within subject, suggesting that for these chemicals, a serum sample provides a reliable monitor of internal, fetal exposure to these estrogenic compounds. Our population also has a very high rate of premature delivery with 10% of births in 2004 occurring before 37 weeks gestation (unpublished local data). Therefore, we sought to evaluate two types of endocrine disruptors, a environmental contaminant that has previously been associated with preterm labor, DDE, and two common dietary derived phytoestrogens, daidzein and genistein. The study was approved by our local Ethics Review Board.

## Methods

Cases were defined as primiparous women having a premature delivery at greater than 23 weeks and less than 35 weeks gestation following spontaneous labor. The gestational age of <35 weeks was chosen as this corresponds to clinically significant premature labor and this definition has been used in many contemporary studies in prematurity [19-23]. Cases were approached on day one postpartum for consent by the study nurse and asked for a serum sample. After recruiting a case, the next 10 women who delivered a term infant, and were within 5 years of age of the case subject, were approached until two controls were recruited. Only primigravid subjects were included in the study in order to avoid the confounding problems that occur in organochlorine exposure from the substantial reduction in total body burden through previous parturition and breast feeding[24,25]. Women with a stillbirth or a fetus with known congenital anomalies were not included. Based on our preliminary data, we intended to recruit 26 cases and 52 control subjects. DDE was extracted from serum by solid phase extraction. Lipids were then removed from the extract and DDE was analyzed by gas chromatography/mass spectrometry (GC/MS). The limit of quantification for DDE was 0.05 ng/ml. Lipid concentrations were determined gravimetrically. Phytoestrogen (daidzein and genistein) conjugates in serum were hydrolyzed by enzymes in helix Pomatia. The compounds were then extracted from the sample with lipids removed. The phytoestrogens were derivatized via trimethylsilylation and analyzed by GC/MS. The limit of quantification for daidzein and genistein was 0.20 ng/ml. Undetectable levels were assigned a value of .001 ng/ml for analysis. We had deuterated internal standards for daidzein and genistein and internal standards for DDE in which the recovery was close to 100%. The sample size was based on a cross sectional survey of women in the second trimester of pregnancy[18]. In that study, the mean serum concentration of daidzein was 4.75 ng/ml, with a standard error of 3.52 (range 0–136.2 ng/ml). Based on these estimates, 12 cases and 24 controls were estimated to be required to have 80% power to detect a 50% difference in the mean serum daidzein levels between the two groups. The analysis was planned in advance. It was anticipated that the levels of DDE and of the phytoestrogens would be skewed and that transformation of the data would be required. Univariate analysis was planned using the student's t-test. Multivariate logistic regression was planned using potential confounders, such as weight and smoking during pregnancy.

## Results

**Figure 1:**
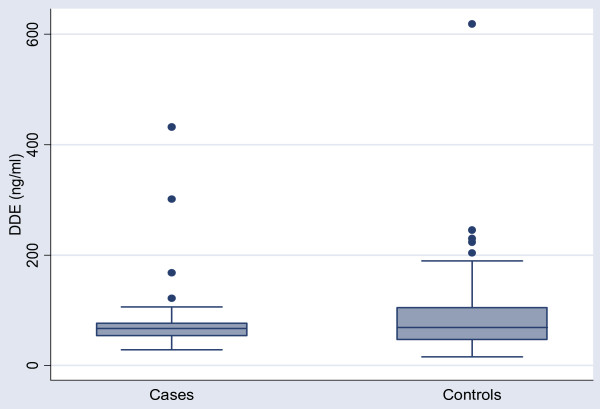
Post partum serum DDE levels (ng/ml) in Cases (n = 26) vs Controls (n = 52).

The demographic characteristics of the subjects were similar and are provided in Table 1. During recruitment only 3 eligible cases declined to participate. The women in the study were born between 1964 and 1983 and were recruited in 2002. DDE levels were corrected for serum lipid concentration. The median corrected DDE level in cases was 67.02 ng/g lipid, range (28.57, 431.88) and in controls was 69.29 ng/g lipid, range (15.79, 618.52). Figure 1. As the DDE levels were positively skewed, they were log transformed for further analysis. The log mean concentration of DDE was similar in the case and the control groups: 4.29 vs 4.32 ng/g lipid p = .85 (see Table 2). Adjusted analysis by multivariate logistic regression with variables for maternal weight, smoking and log DDE also failed to demonstrate an association with premature delivery OR = 1.15 (.47, 2.78).

**Table 1:** Maternal characteristics and pregnancy outcome.

	Cases (n = 26)	Controls (n = 52)
Age	29.2 (19–35)	29.7 (22–38)
Maternal weight (kg)	75.4 (55–130)	77.5 (54–165)
Gestational age at delivery (weeks)	30.5 (24–35)	39.8 (37–42)
Birth weight (gm)	1580 (670–2518)	3817 (2606–4612)
Smoker %(n)	12 (3)	4 (2)

**Table 2:** Serum DDE cases vs controls.

	Cases (n = 26)	Controls (n = 52)	P value
DDE (median, range)	.43 ng/ml (.16, 2.98)	.49 ng/ml (.09, 3.34)	
DDE corrected for lipid (median, range)	67.02 ng/g lipid (28.57, 431.88)	69.29 ng/g lipid (15.79, 618.52)	
Log DDE (mean, SD)	4.29 ng/g lipid (.62)	4.33 ng/g lipid (.66)	.85

**Figure 2:**
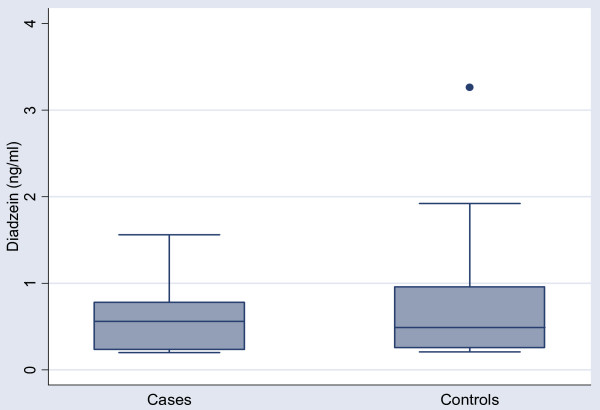
Post partum serum Diadzein levels (ng/ml) in Cases (n = 26) vs Controls (n = 52).

**Figure 3:**
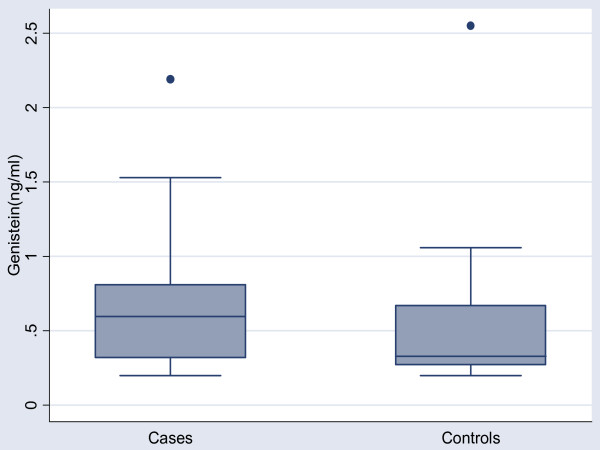
Post partum serum Genistein levels (ng/ml) in Cases (n = 26) vs Controls (n = 52).

In the case group, 13/26 had detectable levels of daidzein (range 0.20 – 1.56 ng/ml) compared to 25/52 controls (range 0.21 – 3.26 ng/ml). Figure 2. For genistein, 14/26 of the case group had detectable levels (range 0.20 – 2.19 ng/ml) compared to 32/52 controls (range 0.21 – 2.55 ng/ml). Figure 3. As this data was also negatively skewed, log transformation was performed, prior to any further analysis. The mean concentration of daidzein was similar in cases and controls: 0.30 vs .34 ng/ml p = 0.91. The mean concentration of genistein was similar in cases and controls: 0.39 vs 0.31 ng/ml, p = 0.61. Adjusted analysis was performed with multivariate logistic regression with variables for log genistein or diadzein levels, maternal weight, and smoking. In this analysis, neither daidzein nor genistein levels was associated with premature delivery: OR = .96 (.81, 1.14), OR = .92 (.77, 1.10) respectively. Addition of both daidzein and genistein together in the model, again, did not suggest any additive or interactive effects, OR = 1.03(.81,1.31), OR = .90(.70,1.15) respectively. Further analysis, including terms for both DDE and daidzein or genistein levels in the same model, again did not show any evidence of additive or interaction effects (data not shown).

## Discussion

Our study did not demonstrate an association between spontaneous premature labor and serum DDE levels. Our results, considered with the results of another case control study on a contemporary population[15], would suggest that DDE exposure is not currently a risk factor for prematurity. One obvious explanation for our results is that, as DDE is no longer in widespread use in North America. Therefore, our subjects are not exposed to the same levels of this toxicant as women delivering in the 1960's who had been the subject of previous studies [14]. In our study, the median DDE concentrations were lower: 0.43 ng/ml range 0.16, 2.98 in cases compared to controls 0.49 ng/ml range 0.09, 3.34. It is also possible that our study was not large enough to detect small differences between the case and control group. Still, a post-hoc power calculation suggests that we had 100% power to detect a 50% increase in DDE levels in cases compared to controls. Our results were also similar to those of the other contemporary case control study[15] in which the median DDE levels were 1.3 ng/ml range 0.5, 17.9 in cases and 1.35 range ng/ml 0.6, 4.0 in controls. However, the levels we observed were substantially lower than those reported by Longnecker et. al., where the majority of normal and preterm birth subjects had concentrations well above 15 ug/l[14]. We, also, reported significantly lower levels of DDE than the Child Health and Development study, in which, the median levels of all subjects was 7 ug/l range 43, 153[26]. Therefore, our results cannot be generalized to regions where subjects can be anticipated as having higher levels of DDE. Furthermore, our study, unlike Longnecker et al[14], included only women with spontaneous premature delivery rather than all premature births of which one third follow induced labor. Indeed, Longnecker's study, also, identified that having a small for gestational age (SGA) fetus was associated with DDE levels, even with the preterm cases removed. It is equally possible that had they excluded induced labor, for which preterm SGA is a common indication, that the association with prematurity would no longer have been evident. Furthermore, our study is the only one we know of that has restricted enrollment to primiparous subjects. As prematurity is associated with primiparity and previous pregnancy and breastfeeding decreases the total body DDE load [26-28] this could have been an unrecognized confounder in previous studies.

Although our previous work had suggested that the placenta and uterus are being exposed to significant levels of the phytoestrogens daidzein and genistein, their levels were not associated with spontaneous premature delivery. One limitation is that we were not able to measure a cumulative exposure to the phytoestrogens and that we had to rely on a single post-partum serum level. It would be difficult and expensive to have performed serial measurements and, as it is difficult to predict the onset of premature labor, even a prospective study would have similar limitations. Genistein and diadzein levels appear to peak approximately 6 hours after intake and their half lives are approximately 6 and 8 hours respectively[29]. Inevitably, the subject's levels will vary depending on their peak level and time from their last ingestion of phytoestrogens. This variability could certainly impair our ability to document any association between premature labor and serum levels. However, it would have been difficult to standardize collection relative to the last phytoestrogen intake. We choose instead to accept this variability and instead recruit patients as soon after delivery as possible. We suspect that even given this variability if phytoestrogens were strongly associated with premature labor we would see indications of this association even with serum samples taken hours after the last intake. However, future investigations should take these factors into consideration with study design.

Like any observational study confounding bias is always a possible explanation for the observed results. We controlled for the two strongest potential confounding factors: primiparity and previous preterm delivery by restriction. Maternal age was controlled for by matching. We also adjusted for other confounders such as maternal weight and smoking in the analysis. We did not however have information on socioeconomic status, a modestly strong risk factor and there is always the possibility that unknown confounders could have affected our results.

As with any negative study, a possible explanation for our results is inadequate power. While we accept this potential criticism, we point out that not only were the differences we observed not statistically significant but that our point estimates were also very close to the null value. However, it is still possible that the effect of these phytoestrogens may be mediated by complex interactions with other estrogenic compounds, as has been indicated in studies using the rat uterotrophic assay [30]. As our study was only able to examine the possible additive effects of DDE and genistein and daidzein we cannot rule out the possible interactions with other agents. This may be a subject for further investigation.

## Conclusion

In this case control study, maternal serum levels of DDE, and the phytoestrogens, daidzein and genistein were not associated with spontaneous premature delivery. There is still a possibility that higher exposure to these substances may contribute to premature labour.

## Abbreviations

DDE (1,1-dichloro-2,2-bis(p-chlorophenyl)ethylene)

DDT (1,1,1-trichloro-2,2-bis(p-chlorophenyl)ethane)

HCG Human Chorionic Gonadotropin

PCB Polychlorinated Biphenyls

## Competing interests

The author(s) declare that they have no competing interests.

## Authors' contributions

All authors contributed to developing the original protocol. CS recruited the patients. SC supervised the analysis of the serum samples. SW performed the analysis. All the authors contributed to and approved the final manuscript.
